# Video-Based Arabic Sign Language Recognition with Mediapipe and Deep Learning Techniques

**DOI:** 10.3390/jimaging12040177

**Published:** 2026-04-20

**Authors:** Dana El-Rushaidat, Nour Almohammad, Raine Yeh, Kinda Fayyad

**Affiliations:** 1 Department of Computer Science, Jordan University of Science and Technology, Irbid 22110, Jordan; naalmohammad24@cit.just.edu.jo (N.A.); kkfayad20@cit.just.edu.jo (K.F.); 2 Google, New York City, NY 10011, USA; raineyeh@google.com

**Keywords:** Sign Language (SL), Arabic Sign Language Recognition (ArSLR), Mediapipe library, Deep Learning, Multimedia

## Abstract

This paper addresses the critical communication barrier experienced by deaf and hearing-impaired individuals in the Arab world through the development of an affordable, video-based Arabic Sign Language (ArSL) recognition system. Designed for broad accessibility, the system eliminates specialized hardware by leveraging standard mobile or laptop cameras. Our methodology employs Mediapipe for real-time extraction of hand, face, and pose landmarks from video streams. These anatomical features are then processed by a hybrid deep learning model integrating Convolutional Neural Networks (CNNs) and Recurrent Neural Networks (RNNs), specifically Bidirectional Long Short-Term Memory (BiLSTM) layers. The CNN component captures spatial features, such as intricate hand shapes and body movements, within individual frames. Concurrently, BiLSTMs model long-term temporal dependencies and motion trajectories across consecutive frames. This integrated CNN-BiLSTM architecture is critical for generating a comprehensive spatiotemporal representation, enabling accurate differentiation of complex signs where meaning relies on both static gestures and dynamic transitions, thus preventing misclassification that CNN-only or RNN-only models would incur. Rigorously evaluated on the author-created JUST-SL dataset and the publicly available KArSL dataset, the system achieved 96% overall accuracy for JUST-SL and an impressive 99% for KArSL. These results demonstrate the system’s superior accuracy compared to previous research, particularly for recognizing full Arabic words, thereby significantly enhancing communication accessibility for the deaf and hearing-impaired community.

## 1. Introduction

Individuals with hearing and speech impairments face significant challenges in communication in their daily lives. Globally, over 5% of the population, approximately 430 million people, including 34 million children, require rehabilitation for debilitating hearing loss. This number is projected to increase dramatically, with more than 700 million people, or 1 in 10 individuals, expected to experience disabling hearing loss by 2050. The situation is particularly critical in the Arab world, where around 38 million people suffer from hearing loss. For this community, sign language serves as their first language and primary communication method. The World Health Organization (WHO) emphasizes the importance of sign language and assistive technologies, such as sign language interpretation, to enhance communication and educational access for those with hearing loss [1].

Sign Language Recognition (SLR) is the process of identifying sign language motions and gestures and translating them into text or speech. However, SLR is considered a challenging task because each sign consists not only of hand shape but can also include contributions from face and body parts. Sign language recognition can be categorized into two types: isolated recognition, which refers to a single hand pose or gesture, and continuous or dynamic recognition, which involves multiple poses corresponding to a word or phrase [2]. Despite the critical need, few people outside the deaf and hearing-impaired community understand sign language, creating a substantial communication barrier. Furthermore, sign languages are not universal; each spoken language often has a corresponding sign language, with variations across countries and regions. For Arabic, with over 200 million speakers across 21 countries, each with numerous dialects, the development of automated sign language recognition is crucial to bridge communication gaps between people.

Previous research on SLR has frequently relied on specialized hardware, such as sensor gloves or Kinect depth cameras [3,4,5]. While these methods can achieve good accuracy, they introduce several practical issues: high cost, limited availability, the need for precise tuning and calibration, and an inability to fully capture comprehensive body postures. Moreover, much of the research in Arabic Sign Language Recognition (ArSLR) has traditionally focused on recognizing individual Arabic alphabet letters or numbers, with limited attention given to the more complex task of recognizing full Arabic words. These limitations highlight a gap in the development of accessible, comprehensive, and high-accuracy ArSLR systems.

Motivated by these challenges, this research aims to develop an affordable, real-time Arabic Sign Language recognition system. Our system leverages standard mobile or laptop cameras, thereby eliminating the need for additional hardware such as gloves, sensors, or specialized cameras. The methodology employs Mediapipe [6], an open-source framework by Google, together with the OpenCV library, an open-sourced computer vision library, for real-time extraction of holistic landmarks, including hand, face, and pose data, from video streams. These extracted anatomical features are then processed by a hybrid deep learning model that integrates Convolutional Neural Networks (CNNs) and Recurrent Neural Networks (RNNs), specifically Bidirectional Long Short-Term Memory (BiLSTM) layers. This CNN-BiLSTM architecture is critical for capturing both spatial features (e.g., hand shapes, body movements within frames) and long-term temporal dependencies (e.g., motion trajectories between frames), which is essential for accurately differentiating complex signs that may appear spatially similar but differ in their dynamic execution, such as the Arabic signs for “eat” and “drink”.

Rigorously evaluated on two datasets—the author-created JUST-SL dataset (21 Arabic words recorded with a mobile phone camera in uncontrolled environments) and the publicly available KArSL dataset [7] (40 Arabic words recorded with a Kinect camera by a professional signer in a controlled environment)—our system achieved 96% overall accuracy for JUST-SL and an impressive 99% for KArSL. These results demonstrate that the proposed system achieves superior accuracy for recognizing full Arabic words compared to previous research, even outperforming studies focused on individual letters. Furthermore, we report on both training and average inference times, highlighting the model’s capability for real-time ArSLR. By providing a robust, highly accurate, and affordable solution, our research significantly enhances communication accessibility for the deaf and hearing-impaired community in the Arab world.

The novelty of this research lies in using the extracted landmarks as input to a CNN-RNN architecture specifically designed and tuned for this study. In addition, we conducted two experiments: Experiment 1: Isolated Feature Model (hands and face), and Experiment 2: Holistic Feature Model (full body). These experiments led to the conclusion that high, yet acceptable, accuracy can be achieved using only the hands and face. Furthermore, the novelty of our work also includes providing a new Arabic Sign Language dataset, namely JUST-SL.

The paper is organized as follows: Section 2 reviews previous work on sign language recognition, focusing on Arabic. Section 3 presents the background, including Mediapipe, deep learning models, and evaluation methods. Section 4 describes the datasets, while Section 5 details the proposed approach. Section 6 reports the experiments and results. Section 7 concludes with contributions and future directions.

## 2. Related Work

### 2.1. Sign Language Recognition Using Specialized Hardware

Early research in SLR often relied on specialized hardware to capture sign movements, such as sensor gloves (also known as data gloves or smart gloves). These gloves are equipped with sensors to trace hand and finger movements, including position, orientation, and motion [8]. Projects like “Talking Hands” used sensor gloves to recognize 24 American Sign Language (ASL) alphabets [3]. Other studies achieved recognition rates of 89% for Malaysian sign language alphabets, numbers, and selected words [9]. For ArSL, smart gloves have been explored with tailored sensor designs to achieve accuracy for various Arabic sign words [10].

Another widely used specialized hardware was the Kinect motion-sensing device [11], which employs a combination of RGB cameras and depth sensors for gesture tracking. Research using Kinect achieved good accuracy, making real-time 3D reconstruction applicable. For instance, one study using Kinect for 1000 ASL phrases reported a highest accuracy of 76.12% for sentence verification [12]. Another applied computer vision algorithms that used Kinect to capture sign characteristics and the depth of the motion, and used an SVM classifier to label gestures for digits from 0 to 9 [13].

Despite achieving good accuracy in some applications, these specialized hardware approaches inherently suffer from several significant limitations. These devices, including sensor gloves and Kinect, are generally expensive and difficult to acquire, posing a barrier to widespread adoption. Furthermore, they necessitate precise tuning and sensor calibration to accurately capture the nuanced finger movements and gestures required for sign language recognition. A critical drawback is their limited scope, as many sign language words involve not only hand gestures but also require specific face and body postures, which these devices may not fully capture, thereby yielding good results only for a restricted range of signs.

### 2.2. Arabic Sign Language Recognition (ArSLR)

Research into Arabic Sign Language Recognition (ArSLR) has seen continuous evolution, progressing from foundational methods to advanced deep learning techniques over an extended period. Early efforts in ArSLR, such as in [14], concentrated on recognizing hand gestures by processing them into characteristics that identified fingertips to achieve a 93.6% accuracy for 30 Arabic alphabet letters. Subsequent advancements included the introduction of polynomial classifiers [15], which notably improved performance by reducing misclassifications by 36% on training data and 57% on test data for Arabic sign language alphabet recognition. The field then saw a shift towards Hidden Markov Models (HMMs), a statistical model widely recognized for its utility in gesture recognition, such as [16], which achieved an overall recognition rate of 82% for isolated ArSL word recognition.

With advances in machine learning and deep learning, more recent efforts have incorporated advanced tools such as convolutional neural networks (CNNs) and recurrent neural networks (RNNs), further improving the capabilities of Arabic sign language recognition systems. In ref. [17], a Convolutional Neural Network was used for the automatic recognition of numbers and letters in the Arabic sign language. They compared their work with traditional approaches based on k-nearest neighbors (KNN) and support vector machines (SVM). In ref. [18], the authors employed transfer learning techniques to achieve automatic recognition of Arabic sign language alphabets using a large dataset containing 54,049 images. They applied various data augmentation and preprocessing techniques and tested multiple transfer learning models. Their approach achieved a training accuracy of 98% and a testing accuracy of 95%. In the work by [19], a novel Faster R-CNN model was designed to extract and map image features while learning the hand’s position in a given image. The proposed approach achieved 93% accuracy on a dataset mainly made up of the Arabic alphabet.

More recently, the Mediapipe 0.10.0 Python library, an open-source framework from Google designed for building multimodal machine learning pipelines (encompassing video, audio, and sensor data), has been increasingly adopted. Mediapipe offers robust hand tracking, face detection, and pose estimation, which are crucial for recognizing the human body holistically in SLR research.

Studies have integrated Mediapipe with deep convolutional neural networks (CNNs) to develop letter recognition systems for ArSL, such as [20] that achieved high accuracies (97.1%) for classifying Arabic alphabets.

Other works have combined Mediapipe’s gesture detection with LSTM for the classification of Arabic letters and words [21], achieving an average performance of 80–85%.

Another approach utilized Mediapipe with a CNN classifier for static gestures and an RNN LSTM for dynamic gestures, reaching 94.4% and 82.7% accuracy, respectively, on a dataset of 10 static words and 10 dynamic words [22].

Additionally, in [23], KNN classifiers were integrated with Mediapipe, achieving a 99.5% accuracy for Quranic Sign Language letters, though this work was limited to fixed images and letters only.

Despite these advancements, existing ArSLR research still faces several critical limitations. A significant portion of previous research, particularly those studies reporting high accuracy, has focused predominantly on recognizing individual Arabic alphabets or numbers, with considerably less attention directed towards the recognition of full Arabic words or continuous sentences. Consequently, when researchers have attempted dynamic sign language recognition involving full words or phrases, the accuracy achieved in earlier studies tends to be lower compared to single-letter recognition. Furthermore, most previous studies do not report training time or inference time, making it challenging to thoroughly assess the real-time applicability and overall efficiency of their models. The lack of availability of publicly accessible datasets for ArSLR remains a challenge as well. Many existing datasets are recorded in controlled, noise-free environments, such as the KArSL dataset, which contrasts with real-world scenarios that introduce confounding factors like noise, varied backgrounds, and inconsistent lighting, all of which can degrade recognition quality. The use of highly complex models, such as Transformer-based architectures, often necessitates significantly larger datasets and substantially higher computational resources, which are not always readily available, especially for systems aiming for real-time performance with limited hardware. Moreover, such models are prone to overfitting on smaller datasets, which is frequently the case with sign language datasets that tend to be limited in size.

Models relying solely on Convolutional Neural Networks (CNNs) or Recurrent Neural Networks (RNNs) have demonstrated limitations in capturing the complex spatiotemporal nuances inherent in sign language. CNNs alone struggle to effectively capture the local spatial features within individual frames, while RNNs alone are not inherently capable of learning features within individual frames, leading to suboptimal performance when spatial dependencies are crucial. This limitation is particularly pronounced for many ArSL signs where meaning is derived from subtle temporal dynamics; for instance, “eat” and “drink” in ArSL may share similar hand movements towards the mouth, but their distinction lies in the hand trajectory (straight versus tilted), making accurate classification impossible for CNN-only models. Additionally, some models struggle to differentiate between signs that exhibit similar hand shapes but vary in subtle body landmarks or temporal trajectory; examples include the words “mom”, “me”, and “today”, due to their shared pointing finger gestures, or the word “rich”, which involves a simple raising fist. This highlights the critical need for a comprehensive spatiotemporal representation that integrates both spatial form and temporal movement for robust recognition.

A summary of the related work for automated Arabic Sign language Recognition, the dataset used, and their achieved accuracies can be found in Table 1. Our method, integrating Mediapipe for feature extractions and a hybrid CNN/RNN model, is listed on the last row of the table.

## 3. Background

In this section, we provide background information for the tools used in the experiments carried out in this research, including Mediapipe and deep learning models, to familiarize the reader with these topics and help them understand the experiments and evaluations.

### 3.1. Mediapipe [24]

Mediapipe, an open-source framework developed by Google, serves as a robust platform for efficiently building and deploying machine learning pipelines. It is particularly adept at enabling computer vision inference through the processing of various sensory data, including image, video, and audio. In the context of Sign Language Recognition (SLR), Mediapipe offers a comprehensive suite of tasks vital for capturing the intricate non-manual features of sign language, such as hand landmark detection, gesture recognition, face landmark detection, and pose landmark detection.

**Figure 1:**
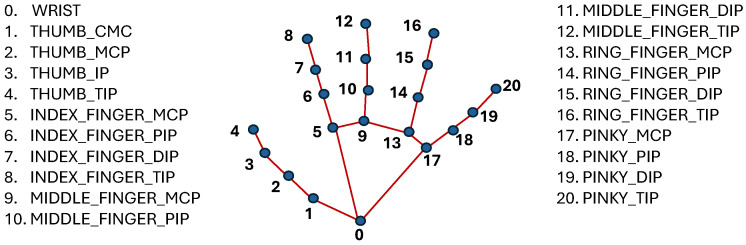
Hand poses recognized using Mediapipe.

**Figure 2:**
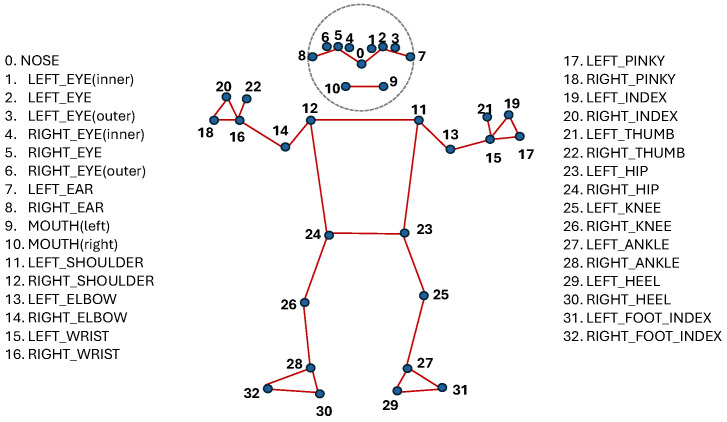
Poselandmark detection guide recognized using Mediapipe.

For this research, we primarily utilize Mediapipe’s Holistic Landmark task. This powerful feature combines pose, face, and hand landmark detection in real time to create a comprehensive map of the human body from a simple video feed. This eliminates the need for specialized hardware like sensor gloves or depth cameras. The Holistic task model outputs a total of 543 distinct landmarks per frame:

1. Hand Landmarks: 21 points for each hand, detailing the position and orientation of the palm, fingers, and thumb. This high level of detail is essential for accurately interpreting handshapes and gestures. Figure 1 shows the hand landmarks and their labels.
2. Face Landmarks: 468 points that map key facial features, allowing for the capture of nuanced expressions that can be crucial in sign language.
3. Pose Landmarks: 33 points that track the position of the head, shoulders, hips, and limbs, capturing the broader body movements that accompany many signs. Figure 2 shows the pose output landmarks and their labels.

**Figure 3:**
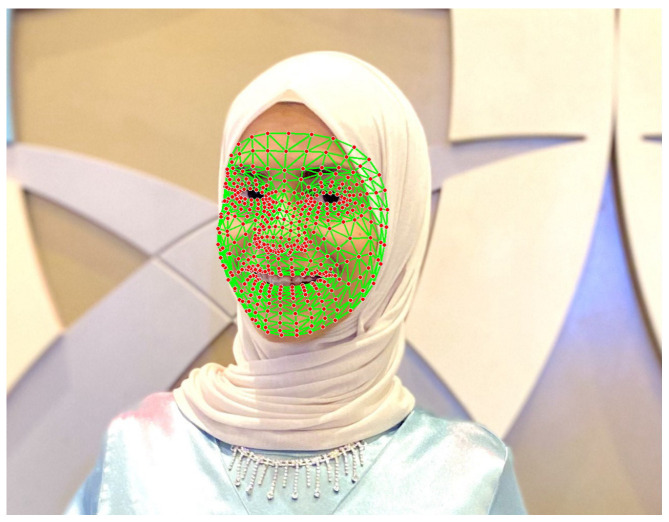
Face landmarks recognized using Mediapipe generated from JUST-SL dataset (red dots are the recognized landmarks and the green lines are the connections (edges) between landmarks).

The face landmark output by Mediapipe is 3-dimensional, blend shape scores (coefficients representing facial expression) to infer detailed facial surfaces in real-time, and transformation matrices to perform the transformations required for effects rendering [25]. Figure 3 shows an example of face landmark output.

By extracting these landmarks, Mediapipe transforms raw video frames into structured numerical data (x, y, and z coordinates for each point), which can then be fed into a deep learning model for analysis and classification.

### 3.2. Deep Learning Models for Spatiotemporal Analysis

Recognizing sign language from video requires a model that can understand not only the specific shapes made by the hands (spatial features) but also the movements and transitions between them over time (temporal features). To achieve this, our study employs a hybrid deep learning architecture that integrates a Convolutional Neural Network (CNN) with a Recurrent Neural Network (RNN), specifically a Long Short-Term Memory (LSTM) network.

1. **Convolutional Neural Network (CNN) for Spatial Feature Extraction**
  A CNN is a deep learning algorithm designed to process and analyze visual data, such as images or individual video frames. Its strength lies in its ability to automatically identify spatial hierarchies of features, making CNN highly effective for object recognition tasks. In the context of SLR, the CNN component of our model processes the landmark data from each frame to learn and recognize key spatial patterns, such as handshapes (e.g., open palm, closed fist) and their orientation.
2. **Recurrent Neural Network (RNN) for Temporal Pattern Recognition**
  An RNN is a specialized architecture built to handle sequential data, such as a time series. Unlike CNN, an RNN maintains an internal memory, allowing it to recognize patterns and relationships across a sequence. We use a specific type of RNN called Long Short-Term Memory (LSTM), which is highly effective at learning long-term dependencies. The LSTM component analyzes the sequence of features extracted by the CNN across multiple frames, enabling it to model the temporal dynamics of a sign, such as the trajectory of a hand gesture.
3. **Hybrid CNN-RNN Architecture**
  Integrating a CNN with an RNN (specifically, a Bi-directional LSTM in our model) creates a powerful system that learns both spatial and temporal features simultaneously. This is critical for distinguishing between signs that may look similar in a single frame but differ in their execution. For example, in the Arabic Sign Language, the signs for “eat” and “drink” both involve a similar handshape moving toward the mouth, but the “eat” sign involves moving the hand in a straight, direct path toward the mouth, whereas for the “drink” sign, the hand is brought to the mouth then makes a tilting motion as if tipping the cup. A CNN alone might struggle to differentiate them. However, the LSTM can analyze the motion across frames and recognize the distinct trajectories between a straight path versus a tilted one that define each sign. This hybrid approach prevents misclassification by building a comprehensive spatiotemporal representation of each sign, making it highly effective for recognizing complete words in a continuous video stream.
4. **Justification of Model Choice**
  While other machine learning models exist, the CNN-RNN architecture was deliberately chosen for its suitability to the task.
  - **Traditional ML Models**
    Traditional ML models, such as SVM or Random Forest, typically struggle with the complex temporal patterns inherent in sign language videos.
  - **Standalone CNN or RNN Models**
    A CNN alone cannot capture temporal dynamics, while an RNN alone is less effective at learning the intricate spatial features within individual frames. The hybrid model overcomes these individual limitations.
  - **Transformer-Based Models**
    While powerful, Transformers generally require vast datasets and significant computational resources, making them less practical for real-time SLR applications with the limited size of available sign language datasets.
  Our chosen architecture provides a balanced and effective solution for accurate, real-time Arabic Sign Language recognition.

### 3.3. Model Evaluation Metrics

To quantitatively assess the performance of our sign language recognition system, we employ a set of standard classification metrics derived from the confusion matrix. The confusion matrix is a table that summarizes the performance of a classification model by comparing its predicted labels against the true labels. It is composed of four key values:

- True Positives (*TP*): The model correctly predicts a sign.
- True Negatives (*TN*): The model correctly identifies that a sample is not a particular sign. (This is more relevant in binary classification; in our multi-class case, it is implicitly distributed among the other correct classifications).
- False Positives (*FP*): The model incorrectly predicts a sign. (e.g., predicts “hello” when the sign was actually “goodbye”). This is also known as a “Type I error”.
- False Negatives (*FN*): The model fails to predict the correct sign. (e.g., predicts “goodbye” when the sign was actually “hello”). This is also known as a “Type II error”.

From these values, we calculate the following metrics to provide a holistic view of the model’s effectiveness:

(1) $$\begin{array}{cc} \mathrm{Accuracy} & =\frac{TP+TN}{TP+TN+FP+FN} \end{array}$$

(2) $$\begin{array}{cc} \mathrm{Precision} & =\frac{TP}{TP+FP} \end{array}$$

(3) $$\begin{array}{cc} \mathrm{Recall} & =\frac{TP}{TP+FN} \end{array}$$

(4) $$\begin{array}{cc} F_{1} & =\frac{2\cdot \mathrm{Precision}\cdot \mathrm{Recall}}{\mathrm{Precision}+\mathrm{Recall}} \end{array}$$

Each measure provides a different intuition for the prediction model. Accuracy is used for balanced datasets, precision is used to avoid false alarms, recall ensures that the true cases are not missed, and the *F*1 score is used for imbalanced datasets or when both precision and recall need to be considered.

## 4. The Datasets

To evaluate the proposed ArSLR system, this study utilized two distinct datasets that represent a spectrum of recording conditions, from controlled laboratory settings to more naturalistic environments. The contrast allows for a comprehensive assessment of the model’s robustness and generalizability.

**Figure 4:**
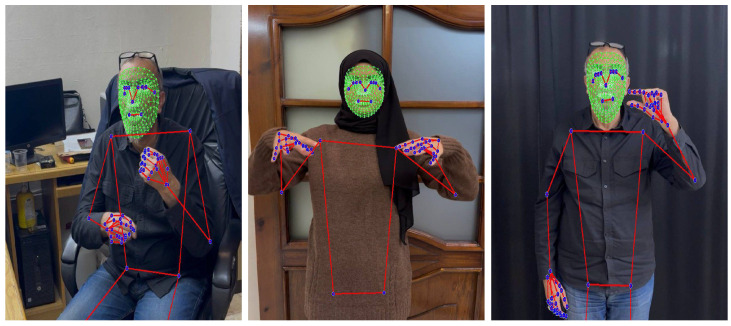
Samples from the JUST-SL dataset. Video has varied backgrounds, lighting, and signers wear different clothing, and can be standing or seated.

1. **JUST-SL dataset** Created by the authors specifically for this research to simulate real-world data collection scenarios, this dataset comprises video recordings of 21 distinct Arabic words, with each word performed 30 times. The recordings feature three different signers, one of whom is a professional. The other signers learned the signs specifically for creating this experimental dataset. The dataset was recorded using an Apple iPhone 11 Pro camera (Apple Inc., Cupertino, CA, USA) with a color resolution of 828 × 1792 pixels at a frame rate of 30 frames per second (FPS). The data were partitioned using a 70% training and 30% testing split at the video level rather than at the frame level. Each recording is approximately 1–3 s long at 30 frames per second. The recordings were conducted in uncontrolled, naturalistic environments, such as workplaces, with varied and dynamic backgrounds. The signers appear in different poses (standing or sitting). The data were captured using a standard mobile phone camera; we deliberately avoided the use of specialized or high-cost equipment. Consequently, the resulting dataset is characterized by environmental noise and variability, presenting a challenging test case for evaluating the recognition system’s performance under non-ideal conditions. Figure 4 shows examples of different signers and backgrounds in the JUST-SL dataset.
2. **The KArSL Dataset** The publicly available King Abdullah Arabic Sign Language (KArSL) dataset [7] was collected under highly controlled laboratory conditions. For this study, 40 Arabic sign language words were selected; each word was performed 40 times by a single professional signer. The dataset was recorded using a Microsoft Kinect V2 sensor, which provides a resolution of 1920 × 1080 pixels, with a frame rate of 30 frames per second (FPS). The dataset documentation does not specify a fixed data partitioning method, allowing researchers to choose a split that suits their task. In our work, we used a 70% training split and 30% testing split at the video level rather than at the frame level. Each video is about 1 s long. The recordings were conducted in a standardized setting, with the signer positioned against a uniform green background and wearing consistent attire. These stringent controls ensure that the data set is clean and free of environmental noise, making it an ideal benchmark to evaluate the maximum potential accuracy of the model when provided with high-quality input.

In Table 2, we discuss the dataset diversity by detailing the number of signers, background conditions, and lighting variations. These factors are further discussed in the Discussion section to clarify how they may influence model generalization and robustness.

## 5. Methodology

This section details the systematic approach taken to develop and evaluate the Arabic Sign Language Recognition (ArSLR) system. The methodology is structured into three primary stages: (1) Data Preprocessing and Feature Extraction, where raw video is converted into a structured format for analysis; (2) Experimental Design, which outlines the two distinct experimental conditions used to test our hypotheses; and (3) Model Architecture and Training, which describes the deep learning framework and the parameters used for training and validation.

### 5.1. Data Preprocessing and Feature Extraction

The initial stage of our pipeline involves extracting features from the video recordings of both the JUST-SL and KArSL datasets suitable for our neural network. This process was applied consistently across all of our video datasets. Much of our image processing was performed using Mediapipe.

1. **Frame Segmentation**: Each video was segmented into a fixed-length sequence of frames. Based on the average duration of a sign, videos from the JUST-SL dataset were converted into 50 frames, while the more concise performed signs in the KArSL dataset were segmented into 30 frames. This is because the KArSL dataset was captured using a Kinect camera and performed by a professional signer, whereas the JUST-SL dataset was recorded with a mobile camera and performed by nonprofessional signers.
2. **Grayscale**: For processing efficiency, we converted all images to grayscale. We found that this does not significantly impact classification results.
3. **Landmark Extraction**: Utilizing the Mediapipe Holistic framework, we extracted a set of 543 landmarks from each frame, with 468 points for face to capture the intricacy of facial expressions, 21 points per hand that capture the palm and each joint of the 5 fingers, and 33 points for the pose that captures the rough posture. The landmarks are output by Mediapipe in XYZ coordinates.

**Figure 5:**
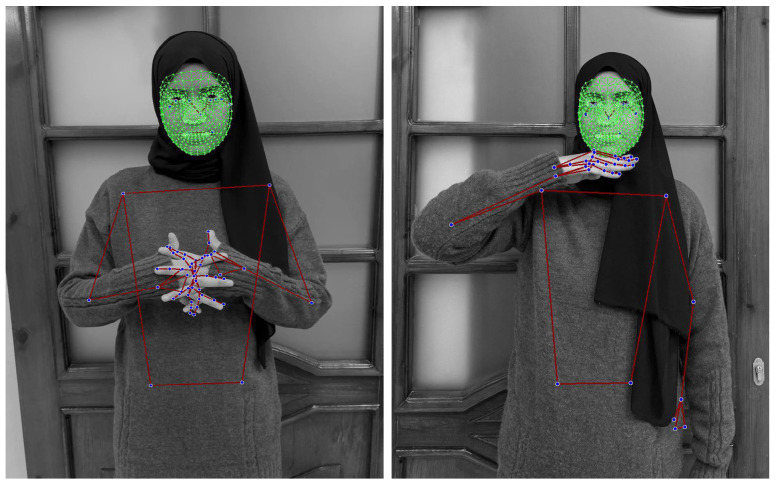
Frames of a sign from the JUST-SL dataset. All landmarks of the face, left and right hands, and posture are shown (dark blue). The face mesh is highlighted in green, while the hands and posture mesh are shown in red, overlaid on the signer.

Figure 5 shows a couple of examples of landmarks extracted from frames of the JUST-SL dataset.

### 5.2. Experiment Setup

To systematically quantify the contribution of different kinematic features to recognition accuracy, a comparative experimental design was implemented. The core objective was to test the hypothesis that including full-body contextual information (e.g., poses) significantly improves the model’s ability to disambiguate signs compared to using only primary articulators (hands and face).

#### 5.2.1. Experiment 1: Isolated Feature Model (Hands and Face)

This experiment was designed to establish a baseline performance by training the model on the most salient features of sign language: hand gestures and facial expressions. This condition tests the sufficiency of these primary articulators for classification accuracy.

We limit the neural network input to the landmark coordinates of just the face, left, and right hands. We leave out the posture landmarks. This effectively reduces the size of the training dataset and allows us to judge the level of accuracy with the limited size of the training data.

Based on our knowledge of Arabic Sign Language, most gestures rely primarily on the face and/or hands. This motivated the design of this experiment, in which we reduced the number of landmarks used during training to make the model more lightweight and efficient. We did not remove any of the images; we only removed posture landmarks, which is straightforward because MediaPipe uses fixed landmark index numbers that clearly specify what each landmark corresponds to.

#### 5.2.2. Experiment 2: Holistic Feature Model (Full Body)

This experiment was designed to assess the performance gain from including secondary, contextual information from body pose. Many signs are differentiated not just by hand shape, but by the hands’ location relative to the torso, shoulder posture, or head tilt. This condition tests whether this additional information resolves ambiguities present in the isolated feature set. In this configuration, the complete and unaltered landmark feature vector of the face, hands, and pose was used directly as input for the model. No features were excluded.

### 5.3. Model Architecture

A hybrid deep learning architecture combining a Convolutional Neural Network (CNN) and a Recurrent Neural Network (RNN) was developed to capture both the spatial and temporal characteristics of sign language. While the core architecture was consistent, specific layer configurations were adapted for each experiment.

The models were trained using Pytorch 2.2 AdamW optimizer algorithm using a categorical cross-entropy loss function, which is appropriate for multi-class classification tasks such as this.

#### 5.3.1. Neural Network Model for Experiment 1

The model for Experiment 1 was designed to process the sequence of isolated face and hand keypoints. It consisted of

- A **1D Convolutional layer** with the Rectified Linear Unit (ReLU) activation function to extract spatial features from the landmark data
- A **MaxPooling layer** that reduces the spatial dimensions of the feature map by taking the maximum value in a sliding window to retain the most important features while making the network more robust to small variations in the input
- A **reshape layer** flattens the feature map into a vector to fit the data into the next layer
- **Long Short-Term Memory (LSTM) layer** to model the temporal sequences of the sign in the forward direction
- A final **dense layer** is added for final classification using the Softmax function to convert the feature map into probabilities for each class. Dropout is applied in between to prevent overfitting.

Table 3 summarizes the neural network layers used in Experiment 1 and the parameters for each layer.

#### 5.3.2. Neural Network Model for Experiment 2

In Experiment 2, we adopt the same data collection methodology as described in Experiment 1, with the exception that no masking was performed to isolate the face and hands. Instead, we utilize the entire frame, incorporating landmarks from the entire body, as shown in Figure 5. No background elements were removed in this setup.

The model for Experiment 2 was engineered to handle the richer, holistic landmark data. It utilized a more complex architecture with 3D Convolutional layers to extract spatio-temporal features directly from the frame sequences. This was followed by multiple Bidirectional LSTM (BiLSTM) layers, which allowed the model to learn temporal dependencies from both forward and backward directions in the sequence, providing a more robust understanding of the gesture’s context.

The model layers are composed of the following:

- **3D Convolutional Layers** to extract spatial and temporal characteristics from the video sequences.
- **MaxPooling3D layers** to reduce the spatial dimensions.
- **Bidirectional LSTM Layers** process the sequential data (temporal features) and capture both past and future dependencies. Batch normalization and dropout are applied to prevent overfitting.
- **Dense Layers:** At the level of the BiLSTM layers, a fully connected dense layer was added for the final classification. After BiLSTM, the last dense layer employs the Softmax activation function to return the final classes.
- **Regularization step** (with the L2 norm) helps reduce overfitting by penalizing large weights.
- The **Optimizer (Adam)** solves for the model weights during training. A low learning rate ($5\times 10^{-5}$) is chosen to ensure fine-tuning.

Table 4 summarizes the neural network layers used in Experiment 2.

**Figure 6:**
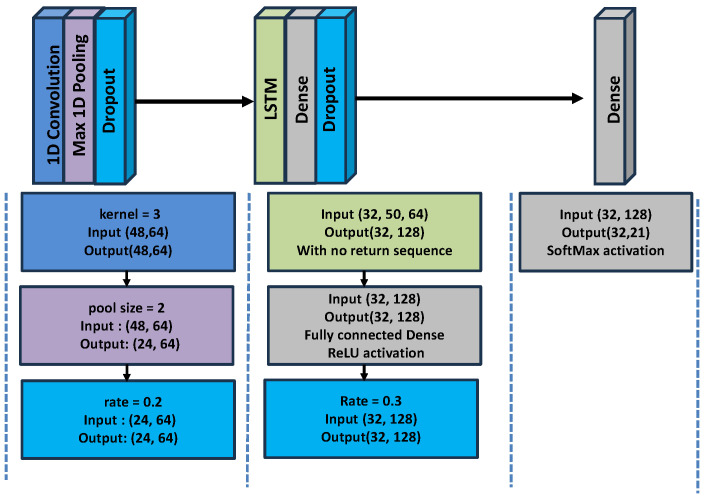
CNN-RNN deep model architecture for experiment 1.

**Figure 7:**
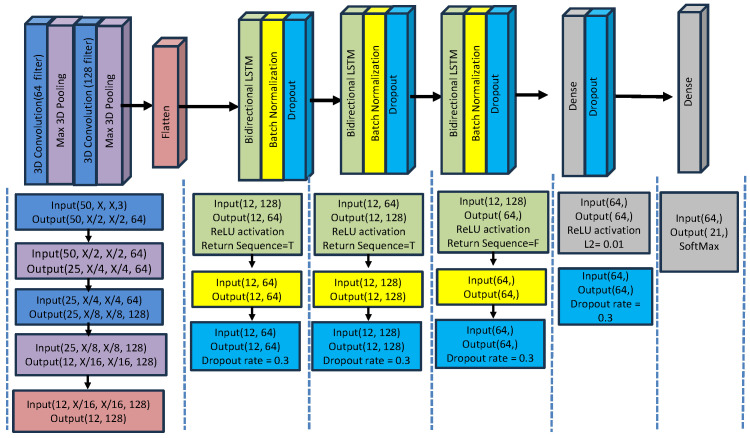
CNN-RNN model architecture for experiment 2.

Figure 6 and Figure 7 provide a summary of the methodology applied in the two experiments, illustrating the architecture of the CNN-RNN model used in each case. The complete set of parameters for the CNN-RNN models is included within the respective figures. Additionally, the figures depict the various layers involved in the training process, which were employed to enable automatic recognition for the two datasets used in this study.

### 5.4. Feature Extraction into the Final Classification Vector

Once the landmark features are extracted, the coordinates are concatenated into the final classification vector. We list the detailed step-by-step process below:

1. Feature Vector Construction We concatenate the four types of landmarks into a singular feature vector:
  - Pose landmarks: 33 points × (x, y, z, visibility) → 132 values.
  - Face landmarks: 468 points × (x, y, z) → 1404 values.
  - Left and right hand landmarks: 21 points × (x, y, z) × 2 → 126 values.
  This results in a total number of 132 + 1404 + 126 = 1662 feature values per frame.
2. Temporal Modeling (Sequence Construction): For each sign video, we select 50 frames that capture the signage. Each frame contributes a 1662-dimensional feature vector, resulting in a sequence of 50 frames × 1662 features = 83,100 feature points per video.
3. Feature Transformation via Deep Learning Layers:
  - Conv3D layers: extract spatio-temporal patterns from the raw feature sequences.
  - Flatten layer: compresses these patterns over each frame.
  - Bidirectional LSTM layers: model the temporal dependencies across frames.
  - Dense layers: reduce the representation into a compact latent space. At the level of the BiLSTM layers, a fully connected dense layer was added for the final classification. After BiLSTM, the last dense layer employs the Softmax activation function to return the final classes.
  - The final Dense softmax layer outputs a probability distribution over the 20 gesture classes.
4. Feature Normalization and Dimensionality Adjustment We performed feature normalization at the Landmark-level and at the level of the CNN layers:
  - Keypoint-level normalization: Normalization was applied at the level of landmark extraction to scaled landmark coordinates in the range of [0, 1]. Mediapipe performs min-max normalization for the xy-coordinates, and relative depth scaling for the z-coordinate, relative to a root joint (like wrist or hips).
  - Batch Normalization: At the level of the CNN layers, batch normalization was applied after each convolutional layer. This accelerates convergence, which is particularly important when training deep CNN-RNN hybrid models. Batch normalization ensures features are well-conditioned before passing them to BiLSTM, which models temporal dependencies. As an example, in Experiment 1, batch normalization was applied with a momentum value of 0.3. This helps normalize the outputs of convolutional layers so that extreme fraction values are reduced toward zero, preventing them from dominating the classification process. Batch normalization was also applied after the RNN layers to stabilize temporal feature learning.
5. Final Classification Output: The output is a 20-dimensional classification vector
  (5) $$\left[p_{1},p_{2},\ldots ,p_{20}\right]\;\mathrm{where}\;\sum_{i=1}^{20}p_{i}=1$$
  and each value corresponds to the predicted probability for a specific gesture.
6. Preservation of Anatomical Relationships: The 1662-dimensional feature vector is organized into a (50, 1662, 1, 3) input tensor to facilitate spatiotemporal learning. In this structure, the three channels correspond to the X, Y, and Z coordinates, allowing the CNN kernels to extract local dependencies between anatomically adjacent joints across the temporal sequence. This representation preserves anatomical relationships by using min-max normalization and relative depth scaling, which ensures that the physical proportions of the signer remain consistent regardless of camera distance or signer posture The proposed representation partially preserves anatomical relationships through the structured ordering of landmarks. Specifically, landmarks belonging to the same anatomical region (e.g., fingers, facial regions, or body joints) are grouped consecutively, maintaining logical proximity in the feature vector. This allows the CNN’s local receptive fields to capture relationships between physically connected or nearby joints.
  However, it is important to note that flattening the landmarks into a one-dimensional vector imposes a linear structure that does not fully reflect the true spatial topology of the human body. While local dependencies are approximated through ordering, explicit graph-based or skeletal representations would more accurately model anatomical connectivity.

**Figure 8:**
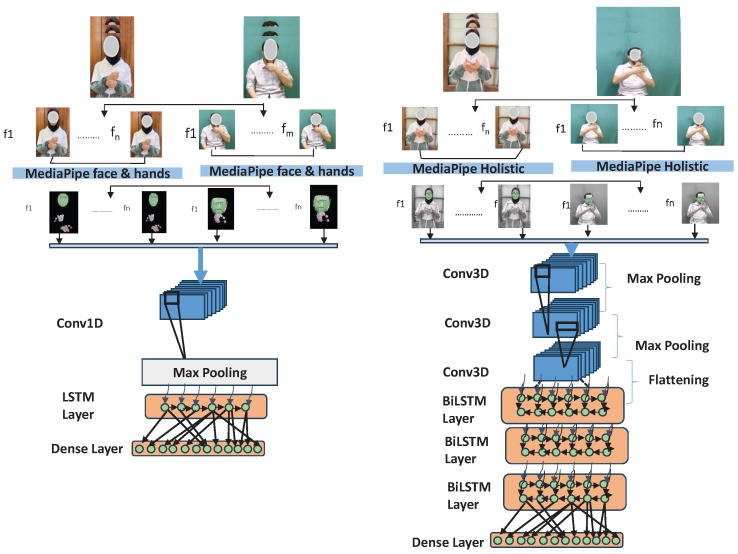
Overviewof training pipeline for Experiment 1 (**left**) and Experiment 2 (**right**). Facial information is masked for privacy protection.

An overviewer of the training pipeline for both experiments can be found in Figure 8. Further details and the corresponding parameters, feature extraction, and classification vectors used in Experiments 1 and 2 are listed in Table 3 and Table 4, respectively.

## 6. Experimental Results

This section presents the empirical results of the two experiments conducted. The findings are organized to first provide a top-level overview of model accuracy performance, followed by a detailed analysis of training dynamics and specific classification errors to interpret the quantitative outcomes. We trained our models with 70% of the dataset and evaluated our methodologies with the other 30%. Our data splitting strategy uses a 70/30 split at the video level rather than at the frame level. This was necessary to ensure that all words in the dataset received an adequate number of videos for training and that no word was excluded from the training process. It is worth mentioning that it is possible that samples from all signers can appear in both training and testing sets.

### 6.1. Overall Model Performance

The primary results of this study, summarized in Table 5, demonstrate the efficacy of our proposed models and confirm the hypothesis that utilizing holistic body features can significantly enhance recognition accuracy.

On the professionally curated KArSL dataset, Experiment 2 (Holistic Features model) achieved a near-perfect accuracy of 99%. This represents a substantial improvement over Experiment 1 (Isolated Features), which itself attained a high accuracy of 94%. This increase underscores the value of contextual body pose information in achieving state-of-the-art performance on clean, high-quality data. A similar trend was observed with the more challenging, naturalistic JUST-SL dataset. The Experiment 2 model achieved an accuracy of 96%, again outperforming the Experiment 1 model’s accuracy of 90%. The consistent performance gain across both datasets provides strong evidence that incorporating pose landmarks is crucial for disambiguating signs, particularly in less controlled, real-world environments.

**Figure 9:**
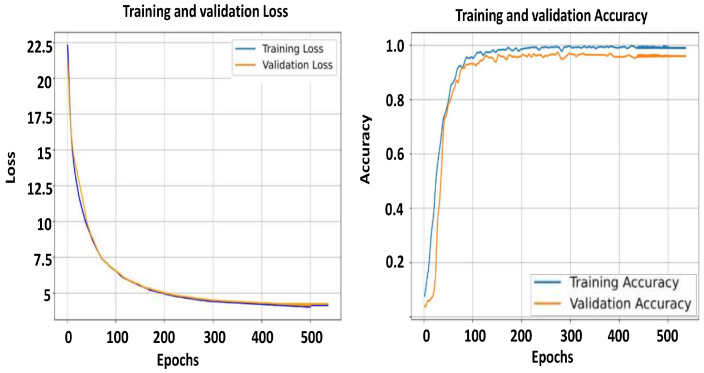
Training and validation accuracy (**left**), training and validation loss (**right**) for the JUST-SL dataset.

**Figure 10:**
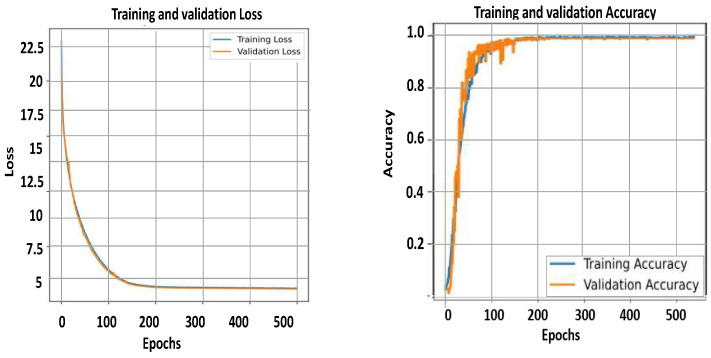
Training and validation accuracy (**left**), training and validation loss (**right**) for the KArSL data.

As observed from the results, the controlled KArSL dataset consistently outperforms the JUST-SL dataset across all experiments. This performance difference can be attributed to the level of dataset diversity, as summarized in Table 2. The JUST-SL dataset exhibits higher variability and noise, as illustrated in Figure 9 and Figure 10, which leads to increased performance fluctuations and slower stabilization during training. These effects are directly explainable by the greater variation and noise present in the JUST-SL dataset, in contrast to the more standardized and consistent nature of the KArSL dataset.

To rule out the possibility that the high accuracies achieved are not due to overfitting, we conducted a detailed sensitivity analysis on both dropout rates (0.3, 0.5, 0.7) and L2 regularization values ($1\times 10^{-4}$, $1\times 10^{-3}$, $1\times 10^{-2}$). The results show a clear and stable trend: low regularization values lead to reduced accuracy due to mild overfitting, while excessively strong regularization (dropout = 0.7 or L2 = $1\times 10^{-2}$) results in underfitting and significantly degraded performance. The optimal performance is consistently achieved with moderate regularization (dropout = 0.5, L2 = $1\times 10^{-4}$), demonstrating that the model behaves robustly across a broad range of hyperparameters rather than relying on a single configuration. These findings confirm that the high reported accuracy is not due to overfitting to a specific setting.

To demonstrate the stability and robustness of the high accuracy achieved in our experiments, we performed a statistical analysis across multiple runs. The results are presented in Table 6. We are listing the mean and the standard deviation for the metric: accuracy, precision, recall, and F1-score. The low standard deviation observed across these runs indicates the robustness and consistency of the proposed model. Moreover, this stable performance is achieved while maintaining a lightweight design and low inference time, as shown previously. These findings highlight both the computational efficiency of our approach and the stability and robustness of the model.

Notably, our approach achieved higher accuracy compared to the most recent studies on Arabic words in [21]. In that study, a custom dataset was used alongside Mediapipe and a CNN model, achieving an accuracy of 83%.

### 6.2. Training Time and Convergence

To emphasize the potential of our model for real-time Arabic Sign Language recognition, we include the average inference time for the test dataset in Table 5. Training was conducted on a 13th-generation Intel Core i5 processor (Intel Corporation, Santa Clara, CA, USA) (2.10 GHz) with 16 GB RAM and no GPU. Since the dataset does not change frequently, training time is less critical than testing time, which is minimal and suitable for real-time applications. Techniques such as pruning could reduce training time; however, investigating these approaches is beyond the scope of the current study. It is worth highlighting that the training time for Experiment 1 was significantly shorter than that of Experiment 2. This difference is attributed to the smaller input data size in Experiment 1, as many unnecessary image details were removed, leaving only the face and hand landmarks. Using KArSL with only Experiment 1, we could achieve a higher accuracy than one of the most recent research in ArSLR [19], which uses only letters, not words. In addition, as shown in Table 5, the average inference time per word is less than 500 ms. This time includes both the processing of the word video into a set of frames and the MediaPipe landmark extraction (labeled in the table as system integration time), as well as the recognition of the extracted MediaPipe landmarks (labeled as methodology time). These two components together constitute the total average inference time per word. The low inference time confirms the model’s suitability for real-time applications. These minimal testing times support the goal of developing an affordable, real-time Arabic Sign Language recognition system that operates on standard hardware. The evaluation was conducted using hardware with limited processing power and no GPU. This efficiency is attributed to the fact that Experiment 1 intentionally reduced the input data size by removing posture landmarks and unnecessary image details, processing only face and hand keypoints. These results demonstrate that high, yet acceptable, accuracy can be achieved using a lightweight and efficient model.

In addition to the accuracy metrics we provide in Figure 9 and Figure 10, we present two plots for the training and the validation accuracy and loss of the two datasets against epoch numbers. The curves reveal the consistent advantage of incorporating CNN and RNN models for both datasets. It is noted last that we cannot reach the high accuracy of any of the datasets from the early epochs; more fluctuation can be seen in the accuracy curve for the KArSL dataset, which might be due to the learning rate starting to stabilize around epoch 200, compared to the JUST-SL dataset in which the curve stays fluctuating until a much later epoch. This is explainable due to the variation in the dataset and the noise available in the JUST-SL dataset compared to the standardized dataset KArSL, which is consistent between the frames and the various repetitions of each word in the dataset. After approximately 200 epochs, we can conclude from the figures that both datasets have reached convergence. The validation accuracy reaches its maximum and then remains stable. This plateau suggests that the model has learned the necessary patterns by epoch 200, and an early stopping strategy could be applied at this point to reduce computational resources and training time. It is worth mentioning that the number of iterations per epoch in the JUST-SL data set is around 600 iterations, while it is around 750 for the KArSL, given that this dataset is considered larger with 40 words compared to 21 words in the JUST-SL dataset.

**Figure 11:**
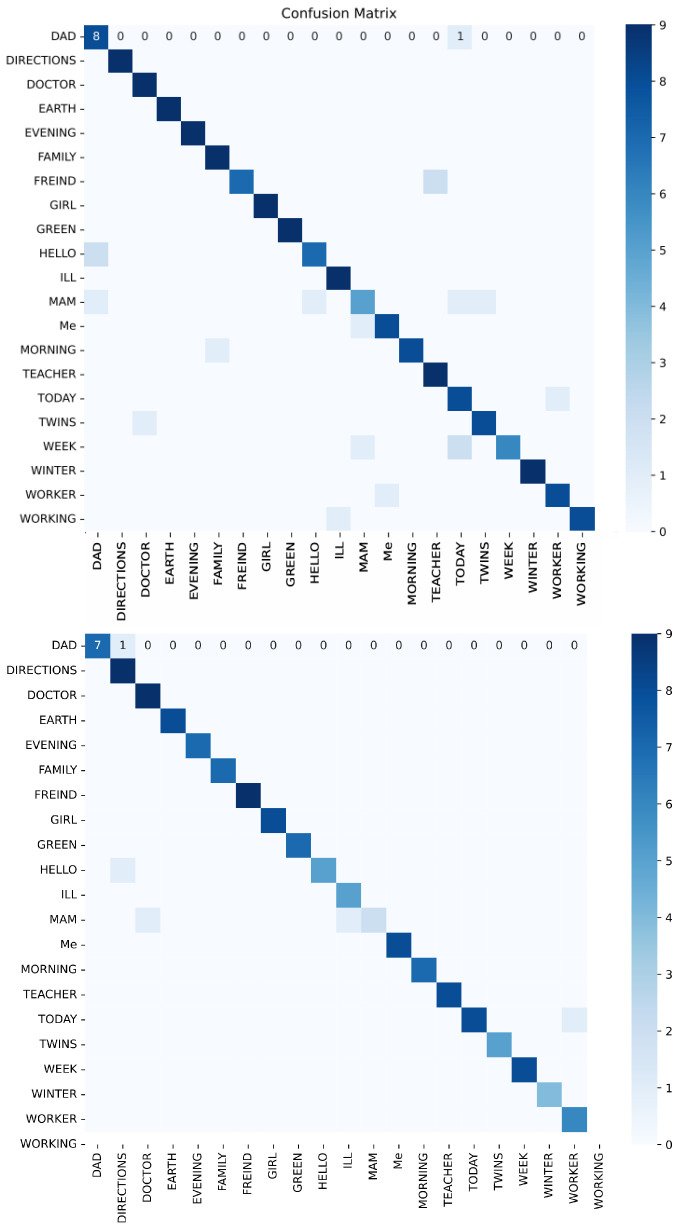
Confusion matrix for Experiment 1 (**top**) and Experiment 2 (**bottom**) of the JUST-SL dataset.

**Figure 12:**
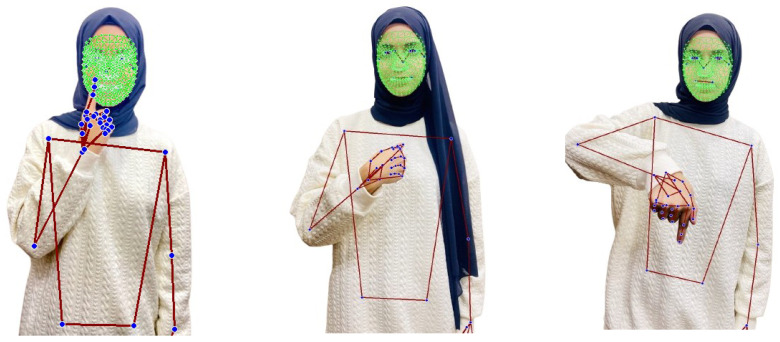
The ArSL signs for the words mom, me, and today (left to right).

As illustrated in the confusion matrix shown in Figure 11, the model demonstrates strong classification performance across most gesture categories, as shown by the strong diagonal and supported by the overall F1-score. However, several systematic misclassification patterns can be observed. The gestures “WINTER,” “WEEK,” and “WORKER” exhibit occasional confusion due to temporal trajectory overlap, as they share similar motion patterns. Additionally, the class “MoM” is sometimes misclassified as “Today” and “ME” due to spatial similarity in hand configuration and position during the initial frames. Overall, no class shows persistent large-scale misclassification, and the remaining errors are localized. An example illustrating misclassification due to finger orientation is shown in Figure 12.

**Figure 13:**
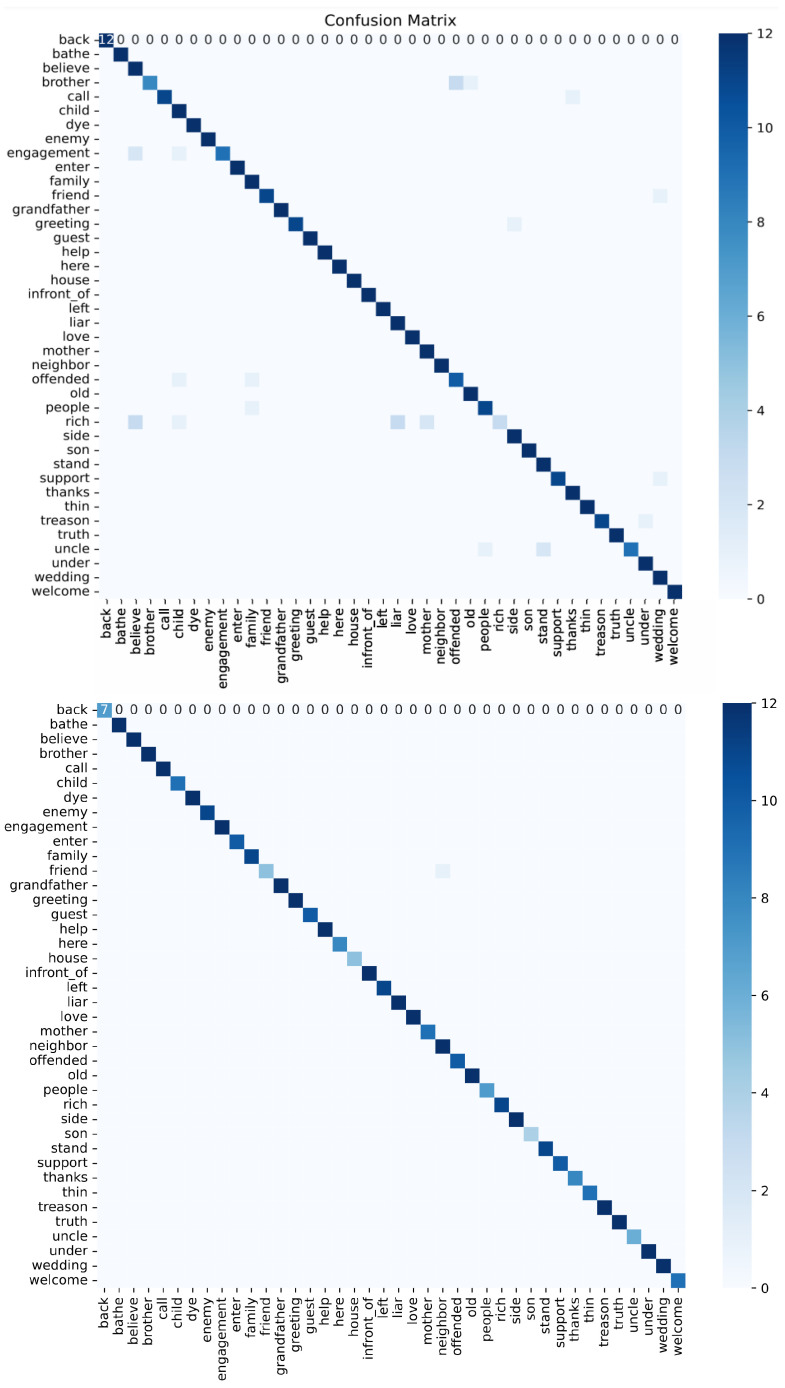
Confusion matrix for Experiment 1 (**top**) and Experiment 2 (**bottom**) of the KArSL data.

**Figure 14:**
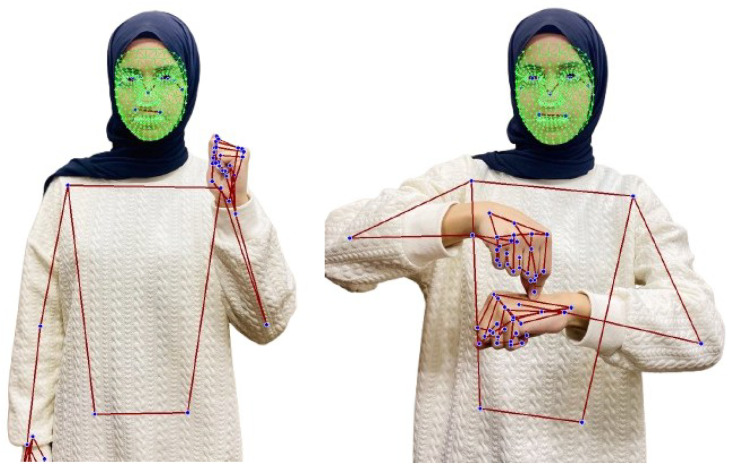
The signs for the words rich (**left**) and people (**right**).

The strong diagonal line in Figure 13 contains high values, indicating high accuracy in gesture recognition. Both experiments show a clear and uninterrupted diagonal, demonstrating that nearly all gesture categories are classified correctly. The observed misclassifications can be categorized into spatial similarity and temporal trajectory overlap. The first group, which includes “rich,” “people,” “child,” “support,” and “greeting,” is mainly affected by spatial similarity, as these gestures share similar hand shapes and configurations, along with overlapping motion characteristics. The second group, which includes “enter,” “engagement,” “offended,” and “welcome,” is primarily influenced by temporal trajectory overlap, as these gestures exhibit similar directional movement and motion patterns, as well as comparable hand placement near the face. An example of misclassification between two gestures with similar hand shapes is shown in Figure 14.

It can also be inferred that in Experiment 2, with the addition of pose landmarks, most of the issues observed in Experiment 1 were resolved, leading to higher prediction accuracy for several gestures. This is evident in both datasets, as shown by the stronger diagonal line in Experiment 2.

Table 7 compares our work with previous studies. Although some models achieved higher accuracy than ours, those models were tested only on Arabic letters. In contrast, when considering models evaluated on both Arabic words and letters, our model achieved the highest accuracy. In addition, we highlighted the real-time classification capability of our approach, whereas other studies did not report either training time or inference time.

### 6.3. Ablation Study

The CNN component is crucial for recognizing the main spatial features of Arabic Sign Language. After removing the CNN layers from the model, the accuracy dropped to 81%, indicating a significant reduction in recognition performance. The BiLSTM component is essential for capturing the temporal features present in the dataset. It is responsible for modeling temporal dependencies and motion trajectories across frames. Without the BiLSTM, the model accuracy decreased to approximately 90%. Each model performs worse when used independently compared to the combined CNN–BiLSTM architecture. This demonstrates that the proposed model is an effective solution for Arabic Sign Language recognition. In addition, we expanded our ablation study to include hand-only, face-only experiments. Our results show that hands-only (48.4%) and face-only (46.3%) perform substantially worse. This indicates that while hand and face landmarks contain most of the information in the sign language recognition, neither of them is enough to recognize the Arabic sign language.

### 6.4. Robustness Analysis

In this subsection, we perform a robustness analysis to evaluate how landmark detection instability affects classification accuracy, to quantify the system’s dependency on Media Pipe extraction quality.

We perform two tests:

- The effect of jittering:

To simulate the effect of jittering, different levels of Gaussian noise were added to the landmarks matrix. We evaluated three noise levels and measured the model accuracy for each case. The resulting accuracies obtained using the updated landmarks matrix are reported in Table 8. The three sigma values used for the Gaussian noise are: low (σ=0.001), medium (σ=0.005), and high (σ=0.01). The results indicate that our proposed model is highly robust to small levels of noise when applied to the landmark coordinates. For the JUST-LS dataset, the accuracy in Experiment 1 remains constant at 90.48% across all tested noise levels, suggesting that the model is largely insensitive to small perturbations in the landmark positions. In Experiment 2, the accuracy slightly decreases as the noise level increases, which indicates moderate sensitivity to higher levels of jittering.

For the KArSL dataset, the model also demonstrates strong robustness. The accuracy decreases slightly in Experiment 2 when noise is introduced, but it remains relatively stable across the tested noise levels. Interestingly, Experiment 1 shows almost no degradation in accuracy, which suggests that small perturbations may act as a form of regularization or noise tolerance during inference.

Overall, the results demonstrate that the proposed approach maintains high accuracy and robustness against moderate levels of Gaussian noise when introduced, which indicates the robustness of the landmarks extraction against camera jittering that could occur in real-world scenarios.

- The effect of missing hand frames:

To simulate missing hand frames, we simulated missing hand frames in the dataset by removing a selected percentage of frames randomly. Three levels of missing data were tested: 5%, 10%, and 20% of the frames. Then we recorded the change in the classification accuracy in Table 9.

For the JUST-LS dataset, the model demonstrates reasonable robustness to small frame losses. When 5% of the frames are removed, the accuracy drops slightly in both experiments, indicating that the model can tolerate minor frame loss. When 10% of the frames are missing, the accuracy decreases further, suggesting that moderate frame loss begins to affect the temporal representation of gestures. With 20% missing frames, the accuracy drops significantly showing that a larger loss of temporal information makes it more difficult for the model to correctly recognize the gestures.

For the KArSL dataset, the effect of missing frames is more pronounced. While the accuracy decreases moderately with 5% missing frames, a substantial drop occurs when larger number of frames are removed, particularly in Experiment 1. This suggests that the recognition performance for this dataset is more sensitive to temporal disruptions.

The results obtained from Table 9 indicate that the proposed model is robust to small levels of frame loss, which may occur due to temporary hand detection failures or occlusions. However, when a large percentage of frames is missing, the accuracy decreases significantly because important temporal cues required for gesture recognition are lost.

- The effect of motion blur:

To further evaluate the robustness of our method under motion blur, we conducted an additional test. We randomly selected 20% of the videos and applied a Gaussian blur with different kernel sizes (3, 5, and 7) to the frames. The blurred frames were then processed using MediaPipe for landmark extraction, and the testing procedure was carried out as usual. The resulting changes in accuracy are reported in Table 10. The results show that the model is highly robust to low to moderate motion blur. Even in the case of a relatively strong blur with a kernel size of 7, the model is still able to maintain high accuracy.

### 6.5. Vocabulary Scalability and Model Complexity

This section provides the necessary discussion on the vocabulary scalability and its effect on the model complexity. We summarize our discussion to the following points:

- Accuracy stability with increasing vocabulary size:
  Our model demonstrates high performance stability as the vocabulary size nearly doubles from the JUST-SL dataset to the KArSL dataset (21 words to 40 words). The accuracy does not degrade when more classes are introduced; instead, it reaches 99.03% on the larger 40-word dataset. This suggests that the hybrid architecture can effectively handle an increasing number of classes without a loss of accuracy.
- Addressing Spatiotemporal Overlap:
  It is expected that as the number of classes increases, an additional challenge arises, namely the likelihood of signs sharing similar trajectories. The bidirectional LSTM layers allow the model to capture motion dependencies from both forward and backward directions.
- Model Complexity and Efficiency:
  We designed our architecture to be lightweight, which allows us to achieve real-time inference of under 500 ms per word even with the limited hardware used. Expanding to a larger vocabulary, such as more than 500 words, may eventually require increasing the capacity of the BiLSTM layers.
  The hybrid architecture remains efficient, achieving real-time inference (under 500 ms per word) even on limited hardware (i5 processor, no GPU). A significantly larger vocabulary (e.g., 500+ words) might necessitate increasing the number of hidden units in the BiLSTM layers. The model currently uses minimal memory and computational resources and can be expanded to include many additional classes without compromising real-time performance.

### 6.6. Limitations

While the results demonstrate promising performance, several limitations must be acknowledged. One of the most challenging limitations is the computational complexity introduced by the deep learning model, especially the LSTM layers. Additionally, when using our SLR system in real-time environments, we may encounter issues related to the various conditions under which the video is captured (e.g., lighting, clothing color, background color, and occlusion). All these factors might affect the quality of the recognition. When using Arabic words, the signs typically involve only one to two movements, which makes the signer’s speed less important. However, when applying our method to sentences containing many continuous signs, the number of frames required to split the videos for recognition could play an important role. We have already faced this issue, which is why we used a different number of frames for each experiment, depending on the speed of the signer. Professional signers tend to make faster movements, so if the frame rate of the video is low, we may not be able to capture the details of the sign performed by the signer.

An important limitation is the lack of publicly available datasets for Arabic sign language. As the size of the training data is a crucial factor in the performance of a neural network, not having access to a larger and more diverse training dataset limited our ability to train and fine-tune our model’s performance and methodology.

The current evaluation protocol utilizes a video-level split that allows samples from the same signers to appear in both training and testing sets. While this ensures that all gesture classes are represented during training, it indicates that the reported accuracies reflect signer-dependent performance. However, we acknowledge that this protocol may not fully capture the model’s ability to generalize to unseen users. Future work will focus on expanding the JUST-SL dataset to include more signers, enabling a subject-independent evaluation where the model is tested on users entirely absent from the training phase.

Finally, we acknowledge the challenges of cross-study benchmarking and cross-dataset validation. While our hybrid CNN-BiLSTM model demonstrates superior performance on the KArSL and JUST-SL datasets, direct comparisons with existing methods are often limited by several factors. First, many prior studies reporting high accuracies focused on recognizing static letters or numbers, which is significantly less complex than recognizing dynamic full words. Second, there is a lack of hardware and environmental alignment across the field; many previous models used specialized equipment such as Kinect sensors, whereas our system achieves high accuracy with standard camera hardware in uncontrolled environments. Furthermore, the absence of standardized, publicly available ArSLR datasets with overlapping vocabularies prevents cross-dataset validation.

## 7. Conclusions and Future Work

This research presents the development of a real-time Arabic Sign Language Recognition (ArSLR) system by integrating CNN and RNN models, leveraging Google’s Mediapipe library for preprocessing. The research utilizes two datasets: the JUST-SL dataset developed by the authors specifically to test our method, and the publicly available KArSL dataset. The generated system is capable of identifying Arabic words with a high accuracy of 99.03% and 96.48% for KArSL and JUST-SL datasets, respectively. The novelty of this research lies in using landmarks extracted with the MediaPipe open-source library as input to a CNN–RNN architecture specifically designed and tuned for this study. In addition, we conducted two experiments: Experiment 1, the Isolated Feature Model (hands and face), and Experiment 2, the Holistic Feature Model (full body). These experiments led to the conclusion that high, yet acceptable, accuracy can be achieved using only the hands and face. Furthermore, the novelty of our work also includes the introduction of a new Arabic Sign Language dataset, namely JUST-SL.

With the support of Mr. Moawia Al-Bzour, we plan to expand the dataset to include more words and phrases, continuing with three signers and collecting data under varied environmental conditions, including different lighting and backgrounds. The expanded dataset will include sufficient videos per signer and cover different dialects, such as Jordanian, Saudi Arabian, and Standard Arabic Sign Language.

As future work, we plan to perform a subject-independent evaluation, which is currently not feasible due to the limited number of videos per signer. This requires increasing the number of videos per signer and resplitting the dataset so that training uses videos from two signers while testing uses videos from the third. The roles of the signers will be rotated, and the average accuracy across experiments will measure the model’s generalization, ensuring no overlap of frames between training and testing.

Expanding the dataset to include all words in the KARSL dataset will also allow testing generalization across datasets, enabling training on one dataset and testing on the other. This is only possible if each word in the first dataset is included in the second, which is part of our future expansion plan. To further improve robustness and enhance cross-signer generalization, we will explore explicit normalization strategies, such as torso-relative alignment.

The expanded dataset will support studies of cross-environment and cross-dialect performance, providing stronger evidence of the model’s generalizability. While this work focuses on isolated-word recognition, the framework can be extended to sentence-level recognition by modeling temporal dependencies across sign sequences.

To demonstrate real-time applicability and promote adoption within the Deaf community, we plan to develop an application programming interface (API) for practical deployment and evaluation. This may require additional Institutional Review Board (IRB) approval for data collection within the community.

## Figures and Tables

**Table 1:** Summary of related work.

Paper	Methods	Dataset	Format	Accuracy
[14]	Features that identified the fingertips	30 Arabic letters	images of bare hands	93.6%
[15]	Polynomial classifiers	30 Arabic letters	images	98%
[16]	Hidden Markov Model	20 isolated Arabic words	images	82%
[17]	CNN model	28 Arabic letters and 11 Arabic numbers	images	90%
[18]	CNN model	32 standard Arabic letters	images	95%
[19]	R-CNN model	28 Arabic letters	mobile-captured images	93%
[20]	Mediapipe and CNN	28 Static hand-sign Arabic letters	images and videos	97%
[21]	Mediapipe and LSTM	44 Arabic words and 6 Arabic digits	videos	85%
[23]	KNN classifier	14 Arabic Quranic letters	images	99.5%
[22]	MediaPipe and CNN/RNN	20 Arabic letters and words	images and videos	94%

**Table 2:** Data diversity for the two datasets.

Characteristic	JUST-SL Dataset (Uncontrolled)	KArSL Dataset (Controlled)
Number of Signers	3 signers	1 signer
Signer Professionalism	1: professional 2: non professional	1: professional
Background and posture Conditions	Variation in background. variation in signer posture.	uniform green background. signer in one posture.
Lighting Variations	variation in lighting	standard lighting
Acquisition Hardware	Commercialized phone: Apple iPhone 11 Pro camera	Specialized capturing hardware: Microsoft Kinect V2 sensor

**Table 3:** Neural Network layers used in Experiment 1.

Layer	Type	Output Shape	Description
Conv1D	Convolution	(48, 64)	Extracts Spatial features using 64 filters, with a kernel of size 3.
Max Pooling 1D	Pooling	(24, 64)	Reduces the feature size by half. Using a pool size of 2.
Dropout	Regulatization	(24, 64)	Applies dropout with a rate of 0.2. To prevent overfitting
LSTM	Recurrent	(32, 128)	LSTM with 128 layers without returning sequences
Dense	Fully conneted	(32, 128)	Dense layer with 128 layers and ReLU activation for feature learning
Dropout	Regulatization	(32, 128)	Applies dropout with a rate of 0.3. To prevent overfitting
Dense	Fully conneted	(32, 21)	Dense layer with 21 layers. Using softmax activation for classification.
Optimizer	Adam W	None	Learning rate = $1\times 10^{-5}$
Epochs	1000	None	None
Batch Size	32	None	None

**Table 4:** Neural Network layers used in Experiment 2.

Layer	Type	Output Shape	Description
Conv3D (64 filters)	Convolution	(50, X/2, X/2, 64)	3 × 3 × 3 filters, ReLU activation. L2 = 0.01. Padding = same
MaxPooling3D	Pooling	(25, X/4, X/4, 64)	Reduces the feature size by half. Padding = same
Conv3D (128 filters)	Convolution	(25, X/8, X/8, 128)	Extracts spatial features. ReLU activation. L2 = 0.01. Padding = same
MaxPooling3D	Pooling	(12, X/16, X/16, 128)	2 × 2 × 2 pool size. Padding = same
Time Distributed Flatten	Flatten	(12, 128)	Flattens each time step.
Bidirectional LSTM	Recurrent	(12, 64)	BiLSTM with 64 layers. ReLU activation. return_sequences = True
Batch Normalization	Normalization	(12, 64)	Normalize the output of the LSTM
Dropout	Regularization	(12, 64)	Prevent overfitting (Dropout rate = 0.3)
Bidirectional LSTM	Recurrent	(12, 128)	BiLSTM with 128 layers. ReLU activation, return_sequences = True
Batch Normalization	Normalization	(12, 128)	Normalize the output of the LSTM
Dropout	Regularization	(12, 128)	Prevent overfitting (Dropout rate = 0.3)
Bidirectional LSTM	Recurrent	−64	BiLSTM with 64 layers. ReLU activation. return_sequences = False
Batch Normalization	Normalization	−64	Normalize the output of the LSTM
Dropout	Regularization	−64	Prevent overfitting (Dropout rate = 0.3)
Dense	Fully Connected	−64	Feature learning. ReLU activation, L2 = 0.01
Dropout	Regularization	−64	Prevent overfitting (Dropout rate = 0.3)
Dense	Fully Connected	(number of actions)	Softmax output for action classification
Optimizer	Adam	None	Learning rate = $5\times 10^{-5}$
Epochs	-	500	Training epochs
Batch Size	None	16	None

**Table 5:** Accuracy measures and training time for the two datasets in both experiments.

Dataset	JUST-LS Dataset		KArSL Dataset	
**Experiment**	**Experiment 1**	**Experiment 2**	**Experiment 1**	**Experiment 2**
Accuracy	90.48%	96.48%	94.38%	99.03%
Precision	91.27%	97.2%	95.28%	99.02%
Recall	90.47%	96.3%	94.37%	99.01%
F1-score	90.36%	96.11%	94.73%	99.04%
Training time	1178.24 s = 19.6 min	464.12 min = 7.4 h	1193.38 s = 19.8 min	2103.72 min = 35 h
Avg. inference time	16.5 s	5 s	12.8 s	5 s
System Integration time	∼25 ms	∼40 ms	∼25 ms	∼35 ms
Methodology time	∼215 ms	∼360 ms	∼205 ms	∼365 ms
Avg. inference time/word	∼240 ms	∼400 ms	∼230 ms	∼400 ms

**Table 6:** Statistical validation of model performance, (mean ± stddev) across multiple runs.

Dataset	Experiment	Accuracy (%)	Precision (%)	Recall (%)	F1-Score (%)
JUST-SL	Experiment 1	90.50 ± 0.26	90.09 ± 1.14	90.49 ± 0.5003	89.45333 ± 0.78
JUST-SL	Experiment 2	95.54 ± 1.33	96.6 ± 0.85	94.65 ± 2.33	94.56 ± 2.19
KARSL	Experiment 1	94.03 ± 0.34	93.99 ± 1.16	94.13 ± 1.03	94.69 ± 0.33
KARSL	Experiment 2	97.8 ± 1.79	90.1 ± 1.428	97.6 ± 2.12	98.02 ± 1.44

**Table 7:** Model accuracy comparison between prior works and our method.

Method	Papers	Dataset Used	Accuracy
Models tested using Arabic letters and numbers			
Polynomial classifier	[15]	Arabic letters	93.55%
R-CNN	[19]	Arabic letters	93%
CNN only	[17,18]	Arabic numbers and letters	95%
KNN calssifier	[23]	Arabic letters	99.5%
Models tested using Arabic words and letters			
HMM classifier	[26]	Arabic words	98.4%
MediaPipe + CNN	[20]	Arabic words and letters	97.1%
Mediapipe + RNN	[21]	Arabic words and letters	85%
Mediapipe + CNN/RNN	[22]	Arabic words and letters	85%
Mediapipe + CNN/RNN	Our work	Arabic words and letters	99%

**Table 8:** Robustness of the proposed method under different levels of Gaussian noise (jittering) applied to the landmark coordinates.

Dataset	JUST-LS Dataset		KArSL Dataset	
**Experiment**	**Experiment 1**	**Experiment 2**	**Experiment 1**	**Experiment 2**
no noise	90.48%	96.48%	94.38%	99.03%
** σ=0.001 **	90.48%	90.72%	93.0%	95.3%
** σ=0.005 **	90.48%	90.3%	94.5%	95.3%
** σ=0.01 **	90.48%	89.9%	94.5%	95.3%

**Table 9:** Robustness of the proposed model under different percentages of missing frames to simulate partial occlusion or hand detection failures.

Dataset	JUST-LS Dataset		KArSL Dataset	
**Experiment**	**Experiment 1**	**Experiment 2**	**Experiment 1**	**Experiment 2**
no missing frames	90.48%	96.48%	94.38%	99.03%
5% missing frames	89.68%	90.1%	87.9%	92.9%
10% missing frames	86.51%	86.9%	63.0%	89.7%
20% missing frames	70.63%	71.2%	25.0%	73.1%

**Table 10:** Robustness of the proposed model under different levels of blurring with different kernels to simulate motion.

Dataset	JUST-LS Dataset		KArSL Dataset	
**Experiment**	**Experiment 1**	**Experiment 2**	**Experiment 1**	**Experiment 2**
No blur	90.48%	96.48%	94.38%	99.03%
Mild blur (filter 3×3)	91.89%	95.20%	93.44%	98.36%
Medium blur (filter 5×5)	91.20%	94.60%	93.44%	98.36%
Strong blur (filter 7×7)	90.60%	93.80%	91.80%	98.36%

## Data Availability

The data presented in this study, JUST-SL, are openly available in GitHub at https://github.com/NOOR-MCS/Justdata26 (accessed on 6 January 2026). The code for reproducing all experiments is available at https://github.com/NOOR-MCS/Sign_language_system (accessed on 6 January 2026).
